# Elevated Heavy Metal(loid) Blood and Feather Concentrations in Wetland Birds from Different Trophic Levels Indicate Exposure to Environmental Pollutants

**DOI:** 10.1007/s00244-024-01085-7

**Published:** 2024-08-12

**Authors:** Dora Bjedov, Jorge Bernal-Alviz, Jorge Andrés Buelvas-Soto, Laura Ana Jurman, José Luis Marrugo-Negrete

**Affiliations:** 1 Centre for Ecology, Evolution and Environmental Changes (cE3c) & CHANGE – Global Change and Sustainability Institute, Faculdade de Ciências, Universidade de Lisboa, Campo Grande, 1749-016 Lisbon, Portugal; 2 Departamento de Biologia Animal, Faculdade de Ciências, Universidade de Lisboa, Campo Grande, 1749-016 Lisbon, Portugal; 3 St. 4A# 3S-81, Jamundí, Colombia; 4 Laboratory of Toxicology and Environmental Management, Department of Chemistry, Water, Applied and Environmental Chemistry Group, Faculty of Basic Sciences, University of Córdoba, Cra. 6 #77-305, Montería, Córdoba, Colombia; 5 PrimeVigilance d.o.o., Oreškovićeva Ulica 20/A, 10020 Zagreb, Croatia

## Abstract

The research assessed the exposure to total mercury (THg), lead (Pb), and arsenic (As) in Colombian wetland species of different trophic levels *Platalea ajaja, Dendrocygna autumnalis* and *Nannopterum brasilianus*. The results show high THg blood levels in *P. ajaja* (811.00 ± 349.60 µg L^–1^) and *N. brasilianus* (209.50 ± 27.92 µg L^–1^) with *P. ajaja* possibly exhibiting adverse effects. Blood Pb concentration was high in *D. autumnalis* (212.00 ± 208.10 µg L^–1^) and above the threshold for adverse effects, suggesting subclinical poisoning. Levels of blood As were below the assumed threshold for detrimental effect (20 μg L^−1^). The mean concentration of feather THg was below the assumed natural background levels (5 µg g^−1^) for all three species. Feather Pb levels exceeded the levels for assumed threshold effects in all sampled *N. brasilianus* (7.40 ± 0.51 µg g^–1^). Results for feather As concentration were below the threshold for adverse impacts in all species, although a positive correlation between As and THg concentrations was detected in *P. ajaja* feathers. The overall results could help understand how metal(loid)s biomagnify through trophic levels and how wetland species may serve as environmental indicators. By exploring the interactions of metal(loid)s within different matrices and body, this study offers insights into the dynamics of contaminant accumulation and distribution in the environment. This concept can be applied to wetlands worldwide, where bird species can serve as indicators of ecosystem health and the presence of contaminants such as heavy metals and metalloids.

**Supplementary Information:**

The online version contains supplementary material available at 10.1007/s00244-024-01085-7.

Heavy metals and metalloids are constituents of the Earth's lithosphere, occurring in variable proportions. Although naturally occurring and ubiquitous, the concentration of heavy metals and metalloids is increasing, due to anthropogenic exploitation causing adverse effects on biota (Espín et al. 2014; Tchounwou et al. 2012). While certain metals such as copper (Cu), iron (Fe), and zinc (Zn) exhibit essential roles within organisms' growth and life cycles, elevated concentrations become toxic (Lucia et al. 2010). In contrast, non-essential metals, including mercury (Hg), lead (Pb), cadmium (Cd), and arsenic (As) lack recognized metabolic functions (Vizuete et al. 2019). Their chronic presence at low levels engenders heightened toxicity, exerting deleterious impacts on living organisms (Kendall and Scanlon 1981; Lucia et al. 2010; Scheuhammer 1987).

Characterized by bioaccumulation, Hg levels increase within trophic webs over time, accentuated by biomagnification processes (Boening 2000). In birds, it is primarily neurotoxic (López-Berenguer et al. 2020), but can affect other physiological aspects (Bjedov et al. 2023a; Evers et al. 2008; Ji et al. 2006; Kobiela et al. 2015; Nicholson and Osborn 1984; Wada et al. 2009). The monitoring of Hg has been previously conducted using non-destructive samples in the blood of coastal waterfowl (Mallory et al. 2018), *Calidris pusilla* (Burger et al. 2018), as well as in feathers of raptor birds (Zolfaghari et al. 2007; Bjedov et al. 2023b). A cumulative toxicant, Pb has diverse pathways of exposure in birds, e.g. inhalation, soil ingestion, and dietary consumption, and it often originates from mining activities or the ingestion of Pb ammunition in hunting scenarios (Franson and Pain 2011; Krone 2018; Levin et al. 2021; Mateo et al. 2001; Pain et al. 2019). For the purpose of environmental monitoring, Pb was analysed in the non-destructive samples, i.e. the blood of *Anas rubripes* (Pain 1989) and *Ciconia ciconia* (Bjedov et al. 2023a), while in feathers it was analysed in *Bubulcus ibis* (Burger et al. 1992) and *Nycticorax nycticorax* (Golden et al. 2009). Distinguished by its diverse chemical forms, As ranges from elemental states to various complexes and causes substantial avian toxicity potential, with accumulation in bedrock and anthropogenic sources exacerbating the exposure pathways (Mateo et al. 2003a, b; Sánchez-Virosta et al. 2015; Tasneem et al. 2020) with the highest concentration in avian fauna on top of the food chain, e.g. birds of prey *Accipiter nisus*, *Tyto alba* and *Falco tinnunculus* (Eisler 2004; Erry et al. 1999; Stohs and Bagchi 1995; Valko et al. 2005).

The issue of heavy metal and metalloid contamination is a matter of substantial significance on a global, regional, and local scale. Within wetland environments, the presence of heavy metal and metalloid contaminants results in various consequences, including the degradation of water quality with subsequent adverse impacts on hydrophytic vegetation and aquatic fauna, thereby culminating in an intricate interplay that contributes to the contraction of avian species diversity within these wetlands (Ali and Khan 2019; Chai et al. 2017; Jovanović et al. 2017; Xia et al. 2021). Colombia, renowned for its exceptional biodiversity and rich wetland avifauna, provides an opportunity to investigate the ecosystem health through the analysis of aquatic avian species, which serve as crucial indicators of wetland health and significant contributors to the functioning of ecosystem processes (Amat and Green 2010; Hartman et al. 2013; Murillo-Pacheco et al. 2018; Xia et al. 2021). Piscivorous and omnivorous avian species inhabiting wetland ecosystems have the potential to serve as valuable indicators for detecting alterations caused by heavy metal and metalloid contamination due to several compelling factors (Xia et al. 2021). Elevated bioaccumulation and/or biomagnification of heavy metals and metalloids within avian species could pose a substantial threat to the reproductive capacity and overall well-being of bird populations (Amat and Green 2010; Frederick et al. 2002).

Given this background and the scarcity of adequate data concerning heavy metal and metalloid concentrations in avian species from different trophic levels within wetlands, this study provides insights into the hazards arising from the presence of these pollutants. To achieve this, non-destructive sampling was performed, i.e. feather plucking and blood sampling. Both feathers and blood have been previously used to monitor heavy metal and metalloids in other avian species and habitats, seeing as these matrices are accessible tissue during ringing, and sampling in parallel with ringing as well as taking morphometric measures reduces the stress of each individual bird. Specifically, our main goals were:

(I) To quantify the levels of Hg, Pb, and As in the blood and feathers of Roseate spoonbill, *Platalea ajaja*, Black-bellied whistling duck, *Dendrocygna autumnalis* and Neotropic cormorant, *Nannopterum brasilianus*, and assess metal(loid) interspecies variability.
(II) To explore the potential relationships between the Hg, Pb, and As concentrations detected in the blood and feathers of the Roseate spoonbill, *P. ajaja*, Black-bellied whistling duck, *D. autumnalis* and Neotropic cormorant, *N. brasilianus* as well as investigate the associations between the Hg, Pb, and As concentrations with the body mass of the *P. ajaja*, *D. autumnalis* and *N. brasilianus*.

## Materials and Methods

### Study Area

**Fig. 1:**
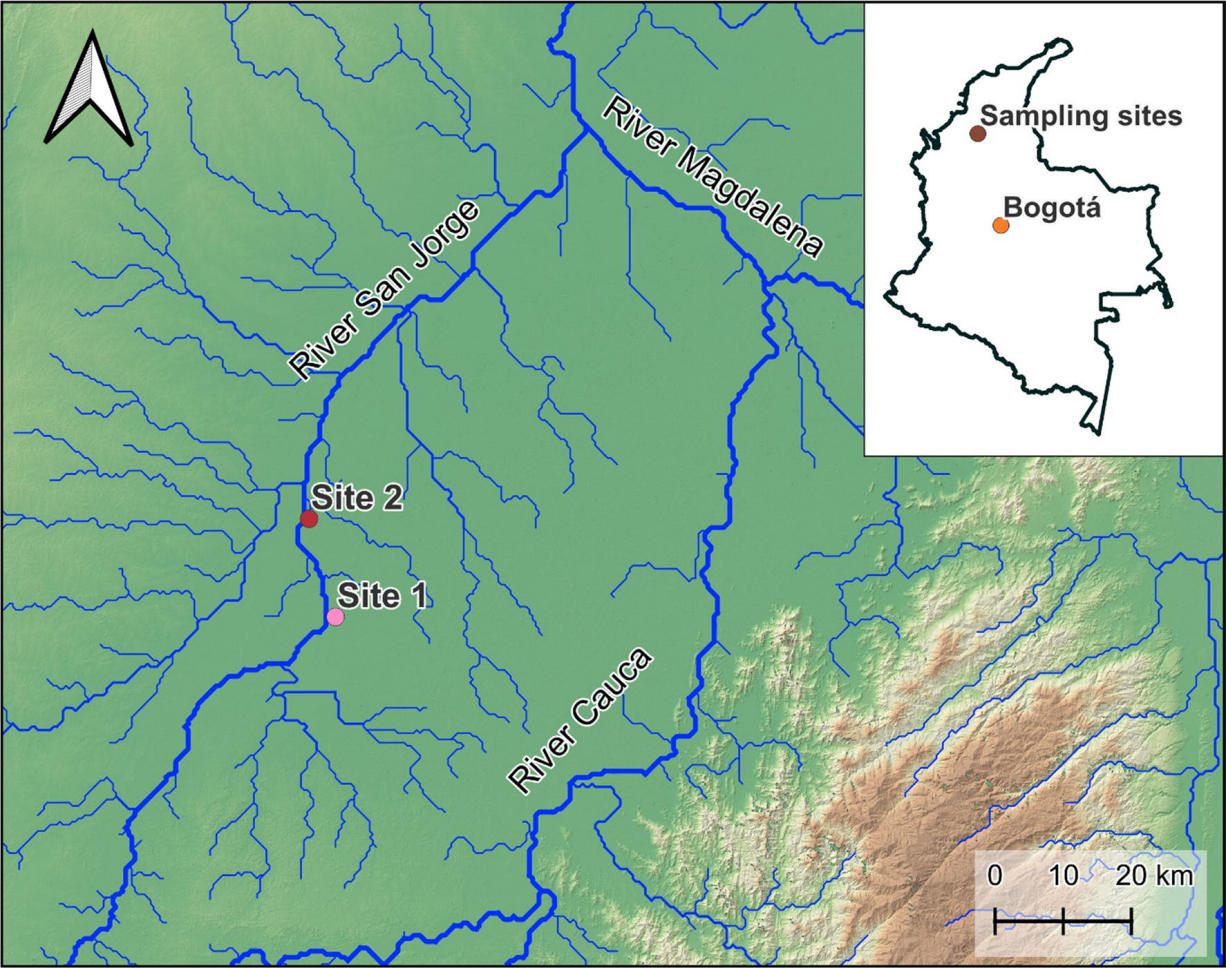
The sampling sites in northern Colombia where blood and feathers were collected from three avian species: Roseate spoonbill, *P. ajaja*, Black-bellied whistling duck, *D. autumnalis* and Neotropic cormorant, *N. brasilianus*

The study was conducted in the Magdalena River basin in northern Colombia, which is recognized as one of the largest tropical rivers globally (Fig. 1). The main sources of contamination in Magdalena River and its tributaries are discharges from industrial mining, use of fertilisers, pesticides and the subsequent runoff, sewerage and wastewater, as well as mining activities and river transport waste from mining in the area’s largest gold mine in San Jorge basin (Salgado et al. 2022). Two sampling sites were located in the area of the San Jorge River: Site 1 (8° 27′34.0" N, 75° 02′ 37.0" W) and Site 2 (8° 35′ 19.3" N, 75° 04′ 43.4" W). Both study sites are in the zone of great impact due to frequent rains and flooding resulting in heavy metal and metalloid accumulation.

### Studied Aquatic Avian Species

The Roseate spoonbill, *P*. *ajaja*, inhabits regions from the Southeastern USA to Argentina (del Hoyo et al. 1992). The species exhibits versatile foraging behaviour in diverse habitats, from hypersaline ponds and marine environments to freshwater lakes. However, their habitat preference leans towards freshwater due to their limited capacity to effectively process hyperosmotic prey. While their primary dietary preference is piscivorous, they also prey on crustaceans, insects, and various other aquatic invertebrates (Britto and Bugoni 2015). The Black-bellied whistling duck, *D. autumnalis*, displays a broad ecological flexibility, nesting in the cavities below the shrub understory and inhabiting ponds with vegetation, e.g. mangrove swamps and cultivated cropland. Their omnivorous diet allows them to exploit a wide range of food sources based on their habitat and seasonal availability. Diet comprises plants, including grasses, aquatic plants, seeds, grains, and crops, and aquatic invertebrates such as insects, crustaceans, and molluscs, which they find in wetland habitats like marshes, ponds, and shallow waters. In addition to aquatic invertebrates, they may forage on terrestrial insects and other small invertebrates when available. Their feeding behaviour is often observed during the night, which is a characteristic of this species (Askin et al. 2019). The Neotropic cormorant, *N. brasilianus* inhabits diverse ecosystems, both freshwater and marine domains. Its dietary preference predominantly centres on small fish present in shallow, sheltered aquatic environments. Foraging habits include a diverse range of tropical habitats, suggesting the potential inclusion of other prey items beyond its prominently favoured small fish, i.e. tadpoles, frogs and aquatic insects (Barquete et al. 2008).

### Blood and Feather Sampling

The field samplings were conducted during the months of April and December 2019, corresponding to the rainy and dry seasons, respectively. The capture methodology employed ten mist nets (measuring 9 m × 2.5 m) strategically deployed in areas identified as crucial transit, feeding, or refuge locations for avian species under study. Capture operations were conducted within a time window spanning from 07:00 to 18:00. In total, 45 adult individuals were sampled, 15 *P. ajaja*, 24 *D. autumnalis*, and six *N. brasilianus*. The assessment of avian body mass was conducted employing an Ohaus CS 2000 digital balance, featuring a precision margin of ± 1.0 g. The execution of the sampling process was carried out in accordance with the permissions and approvals granted by the University of Córdoba and the Regional Autonomous Corporation of La Mojana (CORPOMOJANA). To ensure ethical handling, a veterinarian was instrumental in the sample collection process. Blood samples, ranging from 1 to 3 mL, were drawn from the jugular vein with a sterile needle. Blood samples were then promptly placed into specially prepared tubes containing ethylenediaminetetraacetic (EDTA) anticoagulant acid, and subsequently kept in insulated polystyrene containers supplemented with refrigerant gels, thereby maintaining a consistent storage temperature ranging between 4 and 6 °C (Espín et al. 2021). For feather collection, approximately 10 feathers were obtained from each individual. To ensure methodological rigour, 4–5 contour body feathers were delicately plucked, while another set of 4–5 feathers were extracted from elsewhere. These collected feather samples were sealed within airtight plastic bags, before being subjected to storage (Espín et al. 2021). Subsequent processing of the samples was conducted within the confines of the laboratory facilities belonging to the Water, Applied and Environmental Chemistry research group situated at the University of Córdoba.

### Heavy Metal and Metalloid Analysis

Blood and feather samples from *P. ajaja*, *D. autumnalis* and *N. brasilianus* were analysed in duplicates for heavy metals: total Hg (THg) and Pb. Metalloid As was only analysed in the blood of *D. autumnalis* and *N.* brasilianus and not in *P. ajaja* blood due to insufficient sample volume. Blood samples were put into a Teflon reactor, and then nitric acid (65% HNO_3_) and hydrogen peroxide (30% H_2_O_2_) were added. The proportions of blood, HNO_3_, and H_2_O_2_ were adjusted to a ratio of 1:5:5 based on the volume of blood in the tubes. Subsequently, the mixture was incubated at 90 °C for 24 to 48 h until the digestion of the blood samples was completed. Following digestion, the samples were allowed to cool and adjusted to volumes of 20, 30, or 40 mL using tetra-distilled purified water, depending on the volume of blood intended for analysis. These samples were then transferred to the measuring vessel. For the digestion process, Teflon reactors were prepared by washing them with 3 mL of HNO_3_, followed by two rinses with tetra-distilled water. The reactors were then dried in an oven at 100 °C. The concentrations of As, Hg and Pb in the blood samples were determined using inductively coupled plasma mass spectrometry (ICP-MS) with a PerkinElmer Model Elan 6000 instrument. An analytical quality control programme was implemented. The limits of detection (LOD) for each metal were determined by analysing repeated blanks using the same procedure as for the samples, with the standard deviation (SD) multiplied by three. The limits of quantification (LOQ) for each element, expressed as concentrations in the blood, were calculated based on the mean sample volume analysed. The LOD and LOQ for each metal were as follows: 0.009 μg L^–1^ and 1.21 μg L^–1^ for As, 0.163 μg L^–1^ and 9.49 μg L^–1^ for Hg, and 0.025 μg L^–1^ and 2.69 μg L^–1^ for Pb. Prior to calculating the results, absorbance values from blanks were subtracted. The validity of the method was confirmed by analysing reconstituted lyophilized blood from the certified reference materials Seronorm, Trace Element, Whole Blood 2 (ref. 201,605), and Whole Blood 3 (ref. 102,405) obtained from SERO AS, Norway. The lyophilized blood was reconstituted following the provided instructions. Recovery rates, relative to the concentrations in the reference material, were determined as 106.2% for As, 99.3% for Hg, and 101.8% for Pb.

Prior to analysis, each feather was washed with acetone and deionized water. Subsequently, they were air-dried, weighed, and cut into small pieces using stainless steel scissors. THg concentration in feathers was determined using a DMA-80 Direct Hg analyser (Milestone Italy). To ensure accuracy and precision, a human hair reference (IAEA-086) was tested after every 15 samples. Acceptability criteria were set between 0.53 and 0.61 mg kg^–1^, based on the 95% confidence interval for this standard. The recovery of Hg was determined to be 101.02 ± 6.62%. For Pb and As, feather samples were digested with HNO_3_ following standard methods outlined by the State Health and Family Planning Commission and the State Food and Drug Administration in 2017. Analyses were conducted using the Inductively Coupled Plasma Mass Spectrometer (ICP-MS) (PerkinElmer, USA). Standard curves were generated using a mixed elements standard solution (for As, Pb) with an R-squared value greater than 0.99. Feather samples were analysed, and the mixed standard solution (As = 10 μg L^–1^, Pb = 10 μg L^–1^) was tested every 15 samples to verify that the instrument remained within the specified range. Acceptability criteria were set between 80 and 120% recoveries. The recovery rates were determined 95.73 ± 13.27% for As, and 98.34 ± 6.31% for Pb.

### Statistical Analysis

All statistical computations were executed using R version 4.0.0 (R Core Team 2021) and GraphPad Prism version 8.4.3 (GraphPad Prism Inc., California). Censored data sets, i.e. data containing values below LOD common in environmental contaminant research, were treated as absolute values. For a more comprehensive analysis and discussion of the overall findings, we incorporated data on *D. autumnalis* As concentrations in blood and feathers from a previous study (Buelvas-Soto et al. 2022) into our analysis and interpretation of the results. Preceding the analyses, an examination of the data included outlier detection through boxplots and Cleveland dot plots. If the data did not follow a normal distribution (Shapiro–Wilks test, *P* < 0.05), nonparametric tests were used. To test the potential differences between the THg, Pb and As concentration in blood and As concentration in feathers between the *P. ajaja*, *D. autumnalis* and *N. brasilianus*, a pairwise comparison of the mean rank of each column through the Kruskal–Wallis test was made. Dunn’s *post hoc* test was used to correct for multiple comparisons. Due to insufficient sample volume, As was not analysed in the blood of *P. ajaja*, therefore two-tailed Mann–Whitney U test was applied to test the differences between the As concentration in the blood of *D. autumnalis* and *N. brasilianus*. The differences in THg and Pb concentration in feathers were detected by applying one-way ANOVA, followed by *post hoc* multiple comparisons tests, Tukey adjusted. The associations between the blood and feather THg, Pb, As concentrations and the body mass measurements in species *P. ajaja* and *D. autumnalis* were investigated by applying nonparametric two-tailed Spearman correlation analysis. The relationship between THg, Pb and As concentrations in blood and feathers, with body mass measurements in the *N. brasilianus* species, was explored using a parametric two-tailed Pearson correlation analysis. A correlation matrix was computed with a confidence interval of 95% for the variables. To explore the factors influencing the levels of heavy metals and metalloids in blood and feathers, distinct models were identified for each specific metal(loid) analysed. The selection process employed Akaike's information criterion adjusted for small sample sizes (AIC_C_). These candidate models were constructed to assess the hypothesis related to the variation in metal concentration. This reconstruction was carried out using the *aictab* function from the *AICcmodavg* package. Additionally, a model with no effect (*null* model) was included, providing a valuable baseline for model comparison. The candidate models that provide a reasonable fit to the data while utilizing the simplest possible explanation should be prioritized, thereby reducing the risk of overfitting and enhancing the model's potential for generalization. That being said, the model exhibiting the highest parsimony was determined based on the AIC_C_ value and the AIC_C_ weight (*w*_*i*_). The fixed factors in the analysis were the *Metal(loid) concentration*, *Species*, and *Body Mass*, while the *Individual* factor was treated as a random variable. Statistical significance was considered at 0.05 (α) throughout the study. Due to the magnitude of variability exhibited by the heavy metal and metalloid concentrations in the blood and feather samples, the data set was log_10_-transformed and presented as barplots with standard deviation. This logarithmic transformation was undertaken with the objective of enhancing the visual representation of the data, to achieve greater better interpretability.

## Results

**Fig. 2:**
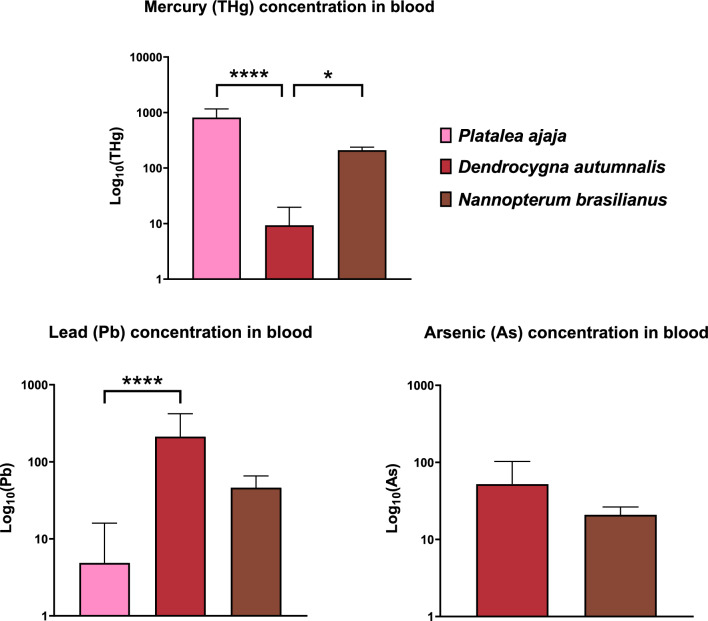
Heavy metals mercury (THg), lead (Pb), and metalloid arsenic (As) concentrations on a log_10_ scale, analysed in the blood of Roseate spoonbill, *P. ajaja*, Black-bellied whistling duck, *D. autumnalis* and Neotropic cormorant, *N. brasilianus* during 2019 from Colombia. Significant differences in the heavy metal and metalloid concentrations between the species are noted with * (*P* < 0.05), **** (*P* < 0.0001)

An overview of the results of THg, Pb and As concentrations in the blood of studied *P. ajaja*, *D. autumnalis* and *N. brasilianus* is shown in Table 1 and Fig. 2. In all analysed samples, THg was detected. Significant differences were detected between the species (*P* < 0.05, Fig. 2). Concentration of THg was significantly lower in the blood of *D. autumnalis* compared to *P. ajaja* (*P*_*adj*_ < 0.0001) and *N. brasilianus* (*P*_*adj*_ = 0.03). Pb concentration was detected in 82% of the samples, with Pb levels below the LOD in eight samples. A significant interspecies variation was observed (*P* < 0.05, Fig. 2); specifically, Pb levels in the blood of *P. ajaja* were significantly lower compared to those in *D. autumnalis* (*P*_*adj*_ < 0.0001). In all samples that were analysed, detectable levels of blood As were found. The findings revealed a lack of significant variation between the species (Fig. 2).

**Table 1:** Descriptive statistics for total mercury (THg), lead (Pb) and arsenic (As) concentrations (μg L^−1^) analysed in blood from three tropical species Roseate spoonbill (*P. ajaja*), Black-belied whistling duck, *D. autumnalis* and Neotropical cormorant, *N. brasilianus* sampled in 2019 from Colombia

	n	Min	25% Percentile	Median	75% Percentile	Max	Range	Mean	SD	Reference
THg (µg L^−1^)										
*Platalea ajaja*	15	470.30	528.10	659.70	1288.00	1439.00	968.60	811.00	± 349.60	This study
*Dendrocygna autumnalis*	24	1.23	4.11	6.77	8.70	42.01	40.78	9.34	± 10.29	This study
*Nannopterum brasilianus*	6	175.40	185.20	204.20	240.40	246.80	71.44	209.50	± 27.92	This study
Pb (µg L^−1^)										
*Platalea ajaja*	15	0.36	0.50	0.50	2.42	40.37	40.01	4.86	± 11.11	This study
*Dendrocygna autumnalis*	24	8.73	101.20	140.50	276.10	929.50	920.80	212.00	± 208.10	This study
*Nannopterum brasilianus*	6	22.15	29.72	43.52	63.11	77.06	54.91	46.16	± 19.45	This study
As (µg L^−1^)										
*Platalea ajaja*	0	N/A	N/A	N/A	N/A	N/A	N/A	N/A	N/A	
*Dendrocygna autumnalis*	6	15.79	15.95	20.09	25.98	28.10	12.31	20.89	± 5.48	Buelvas-Soto et al. 2022
*Nannopterum brasilianus*	24	1.73	6.13	24.14	102.40	143.60	141.80	52.16	± 50.66	This study

**Fig. 3:**
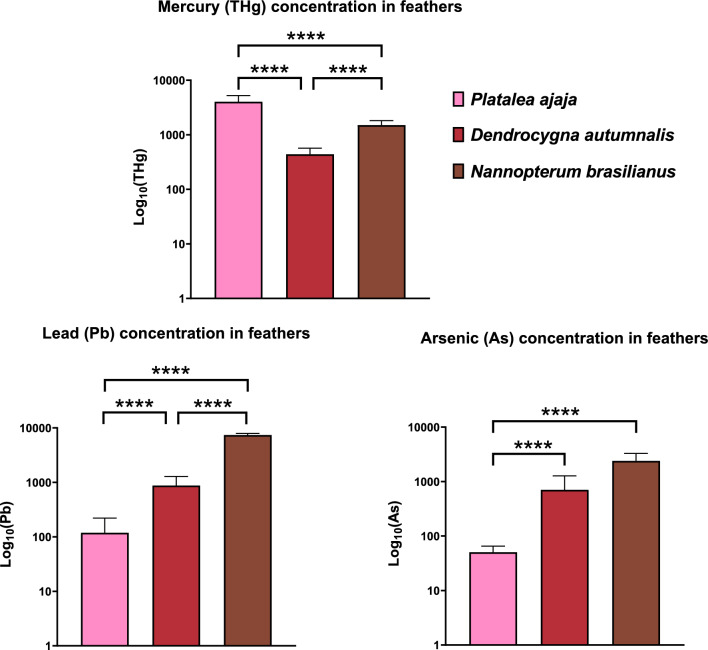
Heavy metals mercury (THg), lead (Pb), and metalloid arsenic (As) concentrations on a log_10_ scale, analysed in feathers of Roseate spoonbill, *P. ajaja*, Black-bellied whistling duck, *D. autumnalis* and Neotropic cormorant, *N. brasilianus* during 2019 from Colombia. Significant differences in the heavy metal and metalloid concentrations between the species are noted with **** (*P* < 0.0001)

An overview of the results of THg, Pb and As concentrations in the feathers of studied *P. ajaja*, *D. autumnalis* and *N. brasilianus* is shown in Table 2 and Fig. 3. In all analysed samples, THg was detected and significant differences were detected between the species (*P* < 0.05, Fig. 3). Concentration of THg was significantly lower in the blood of *D. autumnalis* compared to *P. ajaja* (*P*_*adj*_ < 0.0001, Fig. 3) and *N. brasilianus* (*P*_*adj*_ < 0.0001, Fig. 3). Additionally, THg concentration in the feathers of *P. ajaja* was significantly higher compared to *N. brasilianus* (*P*_*adj*_ = 0.005, Fig. 3). Pb was present in 98% of the analysed samples, with levels below the LOD in only one sample. A significant variation between species was observed (*P* < 0.05, Fig. 3). Specifically, the *N. brasilianus* displayed the highest concentration of Pb in its feathers, a statistically significant increase when compared to both the *D. autumnalis* (*P*_*adj*_ < 0.0001) and the *P. ajaja* (*P*_*adj*_ < 0.0001). Furthermore, the Pb levels in *D. autumnalis* feathers were notably higher in comparison with *P. ajaja* (*P*_*adj*_ < 0.0001). As was detected in all analysed samples and significant variation interspecies was observed (*P* < 0.05, Fig. 3). The *P. ajaja* exhibited the lowest As concentration within feathers, a significant decline when compared with both the *D. autumnalis* (*P*_*adj*_ < 0.0001) and the *N. brasilianus* (*P*_*adj*_ < 0.0001).

**Table 2:** Descriptive statistics for total mercury (THg), lead (Pb) and arsenic (As) concentrations (μg g^−1^) analysed in contour body feathers from three tropical species Roseate spoonbill, *P. ajaja*, Black-belied whistling duck, *D. autumnalis* and Neotropical cormorant, *N. brasilianus* sampled in 2019 from Colombia

	n	Min	25% Percentile	Median	75% Percentile	Max	Range	Mean	SD	Reference
THg (µg g^−1^)										
*Platalea ajaja*	15	2.66	3.06	3.68	4.62	6.17	3.51	4.05	± 1.21	This study
*Dendrocygna autumnalis*	24	0.19	0.36	0.43	0.53	0.68	0.49	0.44	± 0.13	This study
*Nannopterum brasilianus*	6	1.19	1.23	1.40	1.88	1.94	0.75	1.50	± 0.32	This study
Pb (µg g^−1^)										
*Platalea ajaja*	15	0.00	0.06	0.09	0.12	0.42	0.42	0.12	± 0.10	This study
*Dendrocygna autumnalis*	24	0.24	0.53	0.80	1.18	1.64	1.40	0.87	± 0.41	This study
*Nannopterum brasilianus*	6	6.95	7.02	7.25	7.75	8.33	1.38	7.40	± 0.51	This study
As (µg g^−1^)										
*Platalea ajaja*	15	0.03	0.04	0.05	0.06	0.08	0.04	0.05	± 0.02	This study
*Dendrocygna autumnalis*	24	0.21	0.47	0.57	0.76	2.94	2.72	0.71	± 0.57	Buelvas-Soto et al. 2022
*Nannopterum brasilianus*	6	1.12	1.54	2.49	3.16	3.59	2.47	2.40	± 0.89	This study

**Fig. 4:**
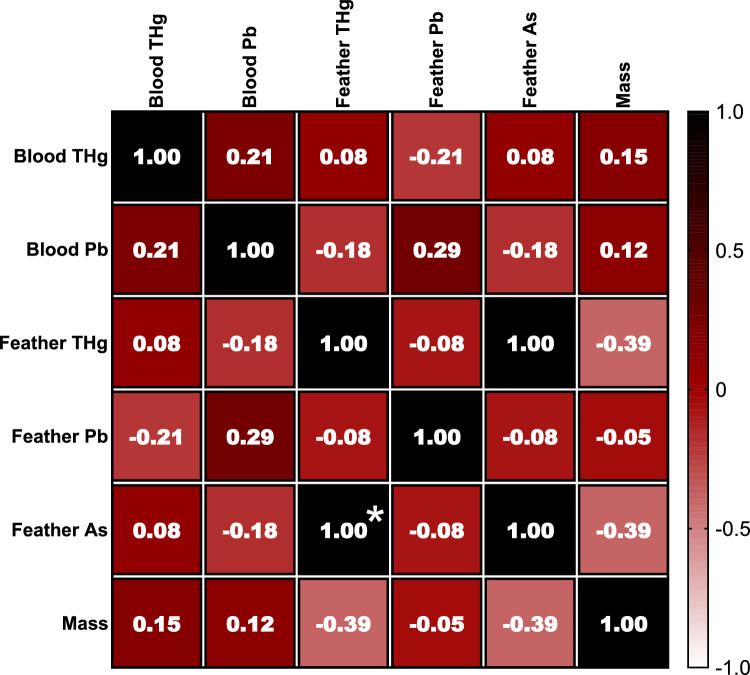
Heatmap of Spearman ranks correlation coefficients (*r*_*s*_) between heavy metals mercury (THg), lead (Pb), and metalloid arsenic (As) analysed in blood and feather with the body mass of Roseate spoonbill, *P. ajaja*. Significant correlation coefficients are noted with * (*P* < 0.05)

Results of the correlation coefficients between the heavy metals and metalloids in different matrices—blood and feathers of *P. ajaja*—are presented in Fig. 4 and Table SI–1. A significant positive correlation was detected in feathers, between As and THg concentration (*P* < 0.0001, Fig. 4).

**Fig. 5:**
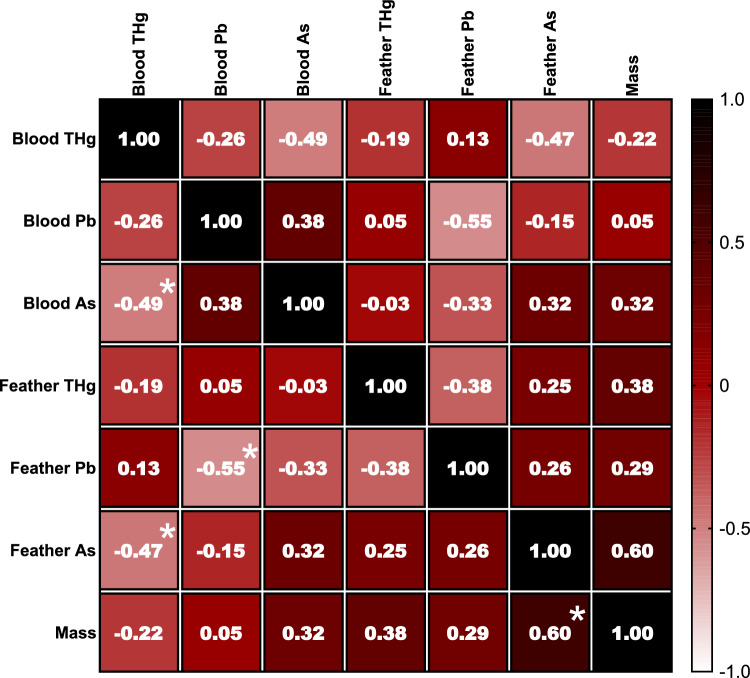
Heatmap of Spearman ranks correlation coefficients (*r*_*s*_) between heavy metals mercury (THg), lead (Pb), and metalloid arsenic (As) analysed in blood and feather with the body mass of Black-bellied whistling duck, *D. autumnalis*. Significant correlation coefficients are noted with * (*P* < 0.05)

Results of the correlation coefficients between the heavy metals and metalloids in different matrices—blood and feathers of *D. autumnalis*—are presented in Fig. 5 and Table SI–2. Significant correlations were detected in blood THg levels, both negative correlations with As in blood (*P* = 0.015, Fig. 5) and feathers (*P* = 0.02, Fig. 5). Negative correlation was observed in Pb levels between blood and feathers (*P* = 0.006, Fig. 5). Positive relationship was recorded between the body mass and As levels in feathers (*P* = 0.001, Fig. 5).

**Fig. 6:**
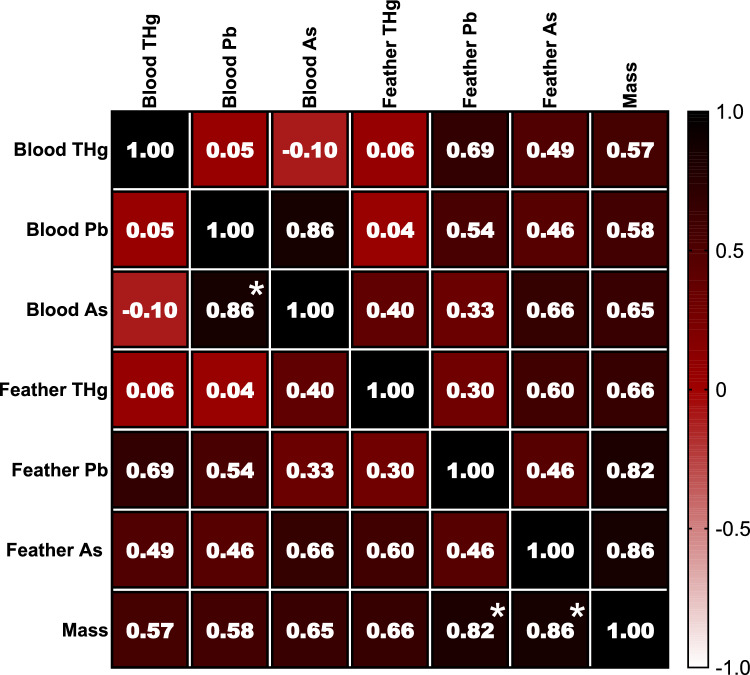
Heatmap of Pearson correlation coefficients (*r*_*s*_) between heavy metals mercury (THg), lead (Pb) and metalloid arsenic (As) analysed in blood and feather with the body mass of Neotropical cormorant, *N. brasilianus*. Significant correlation coefficients are noted with * (*P* < 0.05)

Results of the correlation coefficients between the heavy metals and metalloids in different matrices—blood and feathers of *N. brasilianus*—are presented in Fig. 6 and Table SI–3. A significant positive correlation was detected between As and Pb levels in the blood (*P* = 0.03, Fig. 6). Additionally, two significant positive associations were observed between the body mass and feather Pb levels (*P* = 0.046, Fig. 6) as well as the body mass and feather As levels (*P* = 0.029, Fig. 6).

A comprehensive summary of the prospective models and their respective hierarchical ranking, as determined through the AIC_C_, is presented in Table 3. Distinct patterns in the performance and explanatory power of various models within the analysis were observed. Specifically, we have identified two different scenarios based on the model formulations and their associated AIC_C_ values. The model’s *Blood As*, *Feather THg* and *Feather As* exhibit comparatively lower AIC_C_ values when the model involves the *Species* + *Body Mass* variables. This implies that both the species and the mass of the individual birds jointly contribute to the variation observed in heavy metal and metalloid concentrations, i.e. the inclusion of both factors in the model formulation highlights their shared influence on the levels of the respective metals. On the other hand, the model’s *Blood THg*, *Blood Pb* and *Feather Pb* exhibit lower AIC_C_ values when the coding involves only the *Species* variable. In the context of these specific heavy metal and metalloid concentrations, the *Species* variable alone provides a more parsimonious and effective explanation for the observed variation. For these models, the variation in heavy metal and metalloid concentrations among different species is adequately captured by the *Species* factor without a substantial contribution from the *Body Mass* of the birds.

**Table 3:** The outcomes of model selection involve individual candidate linear mixed-effect models (with the *individual* as a random factor) that potentially provide insight into the observed concentrations of heavy metals and metalloid in the blood and feathers of three bird species: Roseate spoonbill, *P. ajaja*, Black-bellied whistling duck, *D. autumnalis*, and Neotropic cormorant, *N. brasilianus*. The parameters used for evaluation are *K*: a number of estimated parameters; AIC_C_: Akaike’s information criterion value (corrected for small sample size); ΔAIC_C_: difference in AIC_C_ value compared to that of the most parsimonious model; *w*_*i*_: Akaike weight; Res. LL: restricted log-likelihood of each model

Candidate models	*K*	*AIC*_*C*_	*ΔAIC*_*C*_	*w*_*i*_	*Res. LL*
Blood THg ~ 1	3	666.51	82.11	0.00	− 329.96
Blood THg ~ Species	5	584.40	0.00	0.66	−286.43
Blood THg ~ Species + Body Mass	6	585.69	1.29	0.34	−285.74
Blood Pb ~ 1	3	592.52	30.84	0.00	−292.07
Blood Pb ~ Species	5	561.68	0.00	0.89	−275.07
Blood Pb ~ Species + Body Mass	6	565.86	4.18	0.11	−275.83
Blood As ~ 1	3	315.84	7.99	0.01	−154.46
Blood As ~ Species	4	308.40	0.54	0.43	−149.40
Blood As ~ Species + Body Mass	5	307.86	0.00	0.56	−147.68
Feather THg ~ 1	3	812.12	110.07	0.00	−402.78
Feather THg ~ Species	5	705.11	3.07	0.18	−346.81
Feather THg ~ Species + Body Mass	6	702.05	0.00	0.82	−343.95
Feather Pb ~ 1	3	837.42	190.29	0.00	−415.43
Feather Pb ~ Species	5	647.13	0.00	0.68	−317.81
Feather Pb ~ Species + Body Mass	6	648.64	1.00	0.32	−317.24
Feather As ~ 1	3	748.90	69.58	0.00	−371.17
Feather As ~ Species	5	679.51	0.18	0.58	−334.00
Feather As ~ Species + Body Mass	6	679.33	0.00	0.52	−332.59

## Discussion

### Interspecies Variability of Heavy Metal(Loid) Concentrations in Blood and Possible Effects

Environmental exposure to Hg and its effect on avifauna has been previously investigated (Burger and Gochfeld 1997b; Goodale et al. 2008; Jackson et al. 2011; Lavoie et al. 2014; Weech et al. 2006; Whitney and Cristol 2018). Heavy metal Hg is recognized for its propensity to undergo bioaccumulation and biomagnification within aquatic ecosystems, thereby presenting a noteworthy health hazard to piscivorous and omnivorous wetland avian species. The concentration of THg in blood represents recent exposure, primarily via ingestion (Zamora-Arellano et al. 2017). In contrast to our hypothesis that the piscivorous *N. brasilianus* would have the highest THg levels in the blood, *P. ajaja* exhibited the highest THg concentration followed by *N. brasilianus* compared to the *D. autumnalis* (Table 1, Fig. 2). We assume the main difference in blood THg levels between the species are foraging habits and trophic position. *P. ajaja* is a tertiary consumer that forages opportunistically and, therefore, may ingest soil and/or sediment from the bottom of water bodies, subsequently coming into contact with Hg-contaminated particles. This may lead to higher THg exposure compared to *D. autumnalis* which is mostly feeding on plants and invertebrates positioning them as primary and secondary consumers. *N. brasilianus* has significantly higher THg levels in blood compared to *D. autumnalis* (Table 1, Fig. 2) and this may be due to dietary preferences, i.e. *N. brasilianus* is piscivorous which can lead to higher Hg exposure due to biomagnification. Fish tend to accumulate and concentrate Hg as they move up the aquatic food chain (Nyholt et al. 2022). In contrast, *D. autumnalis* might have a diet that includes a larger proportion of plant material or smaller invertebrates with lower Hg content.

Concerning the possible effects, the assumed background THg levels in the blood is 200 µg L^–1^ (Ackerman et al. 2014). The concentration of THg associated with moderate adverse effects is 1000 µg L^–1^ and severe adverse effects in aquatic birds are assumed to be 3000 µg L^–1^ (Ackerman et al. 2014) and 5000 µg L^–1^ has been associated with lower egg and offspring production (Wiemeyer et al. 1984). All levels of THg in *P. ajaja* were above the assumed background levels but below the levels of severe adverse effects. That being said, 27% of the individuals may exhibit moderate adverse effects (≥ 1000 µg L^–1^). Although there is no research on THg levels in the blood of *P. ajaja*, the effects were investigated on other bird species that are ecologically similar and forage in the wetland ecosystems, e.g. *N. nycticorax*, *Egretta thula* (Henny et al. 2007) and *C. ciconia* (Bjedov et al. 2023a; Kamiński et al. 2008). At blood levels of 1000 µg L^–1^, consequences of exposure to Me-Hg include modifications in reproductive behaviours (Frederick and Jayasena 2010; Tartu et al. 2015), breeding success for *Stercorarius maccormicki* (Goutte et al. 2014), decreased egg hatchability, reduction in productivity for *Gavia immer* (Burgess and Meyer 2008), lowered egg hatchability in *Uria lomvia* (Braune et al. 2012), alterations to enzymes associated with glutathione metabolism and antioxidant activity in *Aythya marila*, *Melanitta perspicillata*, *Oxyura jamaicensis* and *C. ciconia* (Hoffman et al. 1998; Bjedov et al. 2023a), as well as compromised behaviour of *G. immer* (Depew et al. 2012). These results indicate the presence of Hg contamination in the areas where *P. ajaja* forage, specifically in the region of the Magdalena River and its tributaries, such as the Jorge River. Anthropogenic activities, including small-scale and artisanal gold extraction operations, may contribute to an increase in environmental Hg levels, as was shown in turtles *Trachemys callirostris* (Zapata et al. 2014). As a consequence of this, a further investigation into potential Hg sources is warranted.

The suggested level of blood Pb indicating subclinical poisoning is > 200 µg L^–1^ (Descalzo et al. 2021; Pain et al. 2019). Reflecting upon this, 67% of the sampled *D. autumnalis* have levels above 200 µg L^–1^ indicating potential adverse effects. This may demonstrate a potential source from the Magdalena River and its tributaries. The toxic profile of Magdalena River sediment was analysed, showing a high Pb concentration (Tejeda-Benitez et al. 2016). The source is presumably from agricultural and industrial activities.

According to Burger and Gochfeld (1997c), the reference value for As-uncontaminated areas is 20 μg L^−1^. The mean blood values observed for both the *D. autumnalis* and *N. brasilianus* exceed the assumed limit of 20 μg L^−1^ (Table 1, Fig. 2). Continuously As-contaminated site reflected a median concentration of 50.50 μg L^−1^ on the blood of *C. ciconia* nestlings (Bjedov et al. 2023a) similar to blood As levels analysed in *N. brasilianus* (Table 1, Fig. 2). It remains inconclusive at this point whether there exists a discernible risk of adverse impacts for the species foraging in the area of River Magdalena and its tributaries. However, if chronic exposure persists over time, the potential for sublethal effects is possible.

### Interspecies Variability of Metal(Loid) Concentrations in Feathers

Analysing feather THg concentration is a frequently used cost-effective tool utilizing non-invasive sampling, and long-term preservation and in addition, can provide historical analysis (Appelquist et al. 1985; Perkins et al. 2020; Rutkowska et al. 2018). Feather THg content has been used to estimate THg body burden (Bjedov et al. 2023b; Kim et al. 1996; Thompson et al. 1991), which can then be associated with feeding location and/or trophic level (Bjedov et al. 2023b; Keller et al. 2014; Ma et al. 2021). Significantly higher THg was observed in *P. ajaja* compared to *N. brasilianus* and *D. autumnalis* (Table 2, Fig. 3). No research information exists on Hg toxicity thresholds for *P. ajaja* with regard to THg concentrations in feathers. Feathers provide a representation of blood THg levels during their formation, and the process of transferring THg into feathers serves as a significant mechanism for eliminating THg from the body (Bottini et al. 2021). It was observed on *Melospiza melodia* (Bottini et al. 2021) and *Sturnus vulgaris* (Carlson et al. 2014) that those growing feathers, while exposed to Hg, exhibited a decline in blood THg levels as the moulting process advanced. Similar trends of blood and feather THg concentration can be observed when compared in *P. ajaja* and *N. brasilianus* (Table 1, Fig. 2, Table 2, Fig. 3). *N. brasilianus* exhibits higher feather THg levels compared to *D. autumnalis* (Table 2, Fig. 3). Feather THg levels were analysed in *N. brasilianus* (Sandoval et al. 2019) and *Phalacrocorax carbo* (Misztal-Szkudlińska et al. 2011), and similar results were obtained when compared with this study (Table 2, Fig. 3). Overall, the mean concentration of feather THg was below the assumed natural background levels (5 µg g^−1^; Burger and Gochfeld 1997a, b, c; Scheuhammer 1991) for all three species, however, in only one individual of *P. ajaja* THg level exceeded the threshold value (6.17 µg g^−1^; Table 2, Fig. 3). It can be concluded that none of the studied species exhibited feather concentrations that exceed the presumed limit for adverse effects (40 µg g^−1^; Sun et al. 2019).

Feather concentrations of Pb were the highest in *N. brasilianus*, compared to both *P. ajaja* and *D. autumnalis* (Table 2, Fig. 3). Additionally, *D. autumnalis* had higher levels of feather Pb levels compared to *P. ajaja* (Table 2, Fig. 3). Compared to other ecologically similar species, feather Pb concentration from *N. brasilianus* had higher levels than *P. carbo* (Mirsanjari et al. 1970), *Bubulcus ibis* (Malik and Zeb 2009) and *Ardea cinerea* (Bjedov et al. 2020). High feather Pb levels in *N. brasilianus* may indicate acute Pb poisoning, i.e. a short-term high-exposure event, and feather Pb concentrations can be used to identify acute poisoning events during the period of feather growth (Vizuete et al. 2019). Assumed feather Pb concentration where poisoning is suspected is usually defined as 4 µg g^−1^ for feathers (Vizuete et al. 2019). It has been shown that adverse effects (negative effects on behaviour, thermoregulation, locomotion, and depth perception resulting in lowered nestling survival) in birds occur if Pb levels exceed 4 µg g^−1^ in feathers (Burger and Gochfeld 1997a, 2000, 2005; Vizuete et al. 2019). For example, some experimental studies have documented adverse effects of Pb in small birds, leading to modification of feather growth rates (10 µg g^−1^ in *Parus major*; Talloen et al. 2008) and altered immune response (20 µg g^−1^ in *Taeniopygia guttata*; Snoeijs et al. 2005). That being said, all of the sampled individuals of *N. brasilianus* exceeded the levels for assumed threshold effects (mean 7.40 µg g^−1^; Table 2). The origin of this contamination could be from the birds ingesting the shot used in shotgun shells (Ancora et al. 2008; Friend et al. 1999) seeing as the consumption-oriented pursuit of introduced animals like cattle, goats, and pigs frequently takes place in the vicinity of lagoons and freshwater regions, i.e. foraging areas of *N. brasilianus*. Research conducted using *T. guttata* through experimental studies has demonstrated that Pb is transferred and deposited onto the feather surface during the process of preening (Dauwe et al. 2002). These findings indicate that the concentrations of Pb in feathers can be influenced by external contamination to a significant extent, rather than solely reflecting the dietary intake of lead during the feather's growth phase. As a result, caution should be exercised when interpreting feather-based results in the context of monitoring dietary Pb exposure.

At elevated concentrations, As can exert adverse effects on reproductive processes (Koivula and Eeva 2010) and function as an indicator of environmental contamination. However, these increased concentrations do not necessarily correspond linearly with internal tissue levels (Geens et al. 2010). Within severely polluted regions, passerine birds have exhibited conspicuous As concentrations reaching up to 30 µg g^−1^ (Janssens et al. 2001). Contrarily, areas devoid of pollution typically display As concentrations below 1 µg g^−1^, while polluted areas tend to manifest levels below 10 µg g^−1^ (Sánchez-Virosta et al. 2015). The present study observed elevated feather-based As concentrations in *D. autumnalis* and *N. brasilianus* relative to *P. ajaja* (Table 2, Fig. 3). Despite overall results below the threshold for adverse impacts across all three species, it is interesting that feather As levels in *D. autumnalis* and *N. brasilianus* surpass those detected in *A. cinerea* feathers from a persistently As-contaminated site (Bjedov et al. 2020).

### Correlation Patterns between Heavy Metal and Metalloids in Different Matrices

We observed a strong positive correlation between As and THg concentrations in *P. ajaja* feathers (Fig. 4, Table SI–1). These findings strongly suggest pronounced biomagnification, given the established tendency of both elements to biomagnify across various trophic levels. Interestingly, our results diverge from those of earlier studies, such as the lack of significant correlations found in the feathers of *Cepphus columba* (Burger et al. 1992), five different seabird species (Furtado et al. 2019), *Somateria mollissima* and *Fratercula cirrhata* (Burger et al. 1992).

On the other hand, a strong negative relationship was detected in blood THg with As levels in the blood and feathers of *D. autumnalis* (Fig. 5, Table SI–2). This negative relationship suggests that there might be some form of interaction or competition between the accumulation or distribution of THg and As in the *D. autumnalis*. THg tends to accumulate more due to biomagnification, whereas As does not biomagnify to the same extent and might be present in different proportions in the diet. That being said, *D. autumnalis* might consume a range of animal prey or plants, each with a different property to accumulate THg and As. Physiological interactions include different biochemical detoxification pathways, i.e. Hg is often bound to proteins such as metallothioneins, while As is detoxified through methylation and subsequent excretion. Additionally, THg and As may exhibit antagonistic interactions within the body meaning the presence of one metal can influence the absorption, distribution, and toxicity of the other. This can occur through competition for binding sites on proteins or enzymes involved in metal detoxification and storage (García-Barrera et al. 2012). Further research is needed to provide insights into how different heavy metals and metalloids are taken up, metabolized, and distributed within the *D. autumnalis*.

A negative association in Pb concentration between blood and feathers was detected, in *D. autumnalis* (Fig. 5, Table SI–2). This negative correlation could indicate a complex relationship between Pb exposure and its distribution within the *D. autumnalis*. It also might suggest that the Pb is being stored or sequestered in the feathers, leading to reduced concentrations in the blood. Such a correlation might have implications for how Pb exposure is metabolized or eliminated within the *D. autumnalis*.

Strong positive correlations were recorded between body mass and feather As concentration in *D. autumnalis* (Fig. 5, Table SI–2), and between body mass, Pb and As feather levels of *N. brasilianus* (Fig. 6, Table SI–3). These results might imply that the accumulation of As in feathers is related to some aspect of the *D. autumnalis* growth or development. Previous research showed As effect on physical development, i.e. *P. major* nestlings exposed to sodium arsenate 0.20 μg g^−1^ d^−1^ had reduced wing growth (Sánchez-Virosta et al. 2018). Furthermore, a positive significant relationship was observed between the concentration of As and Pb in *N. brasilianus* (Fig. 6, Table SI–3). This may suggest their buildup in the blood, however, this relationship needs further study.

## Conclusion

The present study provides the assessment of heavy metal and metalloid concentrations and their interaction within two matrices and body mass, from three wetland bird species from Colombia. The results highlight notable variations in the concentrations of analysed heavy metals and metalloids among bird species at different trophic levels. This emphasizes their unique roles as bioindicators, offering valuable insights into environmental quality and potential risks within their respective habitats. Our results indicate significant changes in analysed heavy metal and metalloid concentrations regarding different bird species and subsequently highlight their distinct roles as bioindicators providing useful information concerning environmental quality and potential hazards within their respective habitats. To conclude, the results of the present study can help in understanding how heavy metals and metalloids biomagnify through food chains and how different wetland species may serve as indicators of environmental quality. By exploring the interactions of heavy metals and metalloids within different matrices and body mass of wetland bird species, the study offers insights into the dynamics of contaminant accumulation and distribution in the environment. This concept can be applied to wetlands worldwide, where bird species can serve as indicators of ecosystem health and the presence of contaminants such as heavy metals and metalloids. Further research involving physiological response is required for a comprehensive assessment to elucidate the extent to which the homeostasis of wild birds is affected. Ultimately, bioindicator species provide a comprehensive review of environmental health and aid in sustainable conservation and management practices. Continuous monitoring of heavy metals and metalloids in birds of Colombian wetlands could ascertain the long-term effects on their survival, overall fitness and habitat health. This recommendation could be extended to wetlands worldwide to ensure the ongoing health and sustainability of these ecosystems in the face of environmental challenges.

## Supplementary Information

Below is the link to the electronic supplementary material.

Supplementary file1 (DOCX 25 KB)

## Data Availability

Data are available on request from the authors.

## References

[CR1] Ackerman JT, Eagles-Smith CA, Heinz G, de la Cruz SEW, Takekawa JY, Miles AK, Adelsbach TL, Herzog MP, Bluso-Demers JD, Demers SA, Herring G, Hoffman DJ, Hartman CA, Willacker JJ, Suchanek TH, Schwarzbach SE, Maurer TC (2014) Mercury in birds of San Francisco Bay-Delta, California: trophic pathways, bioaccumulation, and ecotoxicological risk to avian reproduction. Open-File Report. 10.3133/OFR20141251 10.3133/OFR20141251

[CR2] Ali H, Khan E (2019) Trophic transfer, bioaccumulation, and biomagnification of non-essential hazardous heavy metals and metalloids in food chains/webs—concepts and implications for wildlife and human health. HERA 25(6):1353–1376. 10.1080/10807039.2018.1469398 10.1080/10807039.2018.1469398

[CR3] Amat JA, Green AJ (2010) Waterbirds as bioindicators of environmental conditions. In: Hurford C, Schneider M, Cowx I (eds) Conservation monitoring in freshwater habitats. Springer, Dordrecht. 10.1007/978-1-4020-9278-7_5

[CR4] Ancora S, Bianchi N, Leonzio C, Renzoni A (2008) Heavy metals in flamingos (*Phoenicopterus ruber*) from Italian wetlands: The problem of ingestion of lead shot. Environ Res 107(2):229–236. 10.1016/j.envres.2008.02.004 18359016 10.1016/j.envres.2008.02.004

[CR5] Appelquist H, Drabæk I, Asbirk S (1985) Variation in mercury content of Guillemot feathers over 150 years. Mar Pollut Bull 16(6):244–248. 10.1016/0025-326X(85)90509-0 10.1016/0025-326X(85)90509-0

[CR6] Askin SE, Balkcom GD, Benedict RJ, Rader JA, James JD, Collier BA, Chamberlain MJ (2019) Survival and distribution of black-bellied whistling-duck (*Dendrocygna autumnalis*) in the Southeastern United States. SEAFWA 6:123–128

[CR7] Baos R, Blas J, Bortolotti GR, Marchant TA, Hiraldo F (2006) Adrenocortical response to stress and thyroid hormone status in free-living nestling white storks (*Ciconia ciconia*) exposed to heavy metal and arsenic contamination. HEP 114(10):1497–1501. 10.1289/ehp.9099 10.1289/ehp.9099 PMC1626439 17035132

[CR8] Barquete V, Bugoni L, Vooren CM (2008) Diet of Neotropic cormorant (*Phalacrocorax brasilianus*) in an estuarine environment. Mar Biol 153(3):431–443. 10.1007/s00227-007-0824-8 10.1007/s00227-007-0824-8

[CR9] Bjedov D, Mikuška A, Velki M, Lončarić Z, Mikuška T (2020) The first analysis of heavy metals in the Grey Heron *Ardea cinerea* feathers from the Croatian colonies. Larus Godišnjak Zavoda Za Ornitologiju Hrvatske Akademije Znanosti i Umjetnosti 55:7–25. 10.21857/YPN4OC1KD9 10.21857/YPN4OC1KD9

[CR10] Bjedov D, Velki M, Toth L, Filipović Marijić V, Mikuška T, Jurinović L, Ečimović S, Turić N, Lončarić Z, Šariri S, Al Marsoomi Y, Mikuška A (2023a) Heavy metal(loid) effect on multi-biomarker responses in apex predator: Novel assays in the monitoring of white stork nestlings. Environ Pollut 324:121398. 10.1016/j.envpol.2023.121398 36878276 10.1016/j.envpol.2023.121398

[CR11] Bjedov D, Mikuska A, Begović L, Bollinger E, Bustnes JO, Deme T, Mikuška T, Morocz A, Schulz R, Søndergaard J, Eulaers I (2023b) Effects of white-tailed eagle (*Haliaeetus albicilla*) nestling diet on mercury exposure dynamics in Kopački rit Nature Park. Croatia Environ Pollut. 10.1016/j.envpol.2023.122377 10.1016/j.envpol.2023.122377 37586682

[CR12] Boening DW (2000) Ecological effects, transport, and fate of mercury: a general review. Chemosphere 40(12):1335–1351. 10.1016/S0045-6535(99)00283-0 10789973 10.1016/S0045-6535(99)00283-0

[CR13] Bottini CLJ, MacDougall-Shackleton SA, Branfireun BA, Hobson KA (2021) Feathers accurately reflect blood mercury at time of feather growth in a songbird. Sci Total Environ 775:145739. 10.1016/J.SCITOTENV.2021.145739 33621875 10.1016/J.SCITOTENV.2021.145739

[CR14] Braune BM, Scheuhammer AM, Crump D, Jones S, Porter E, Bond D (2012) Toxicity of methylmercury injected into eggs of thick-billed murres and arctic terns. Ecotoxicology 21(8):2143–2152. 10.1007/s10646-012-0967-3 22760665 10.1007/s10646-012-0967-3

[CR15] Britto VO, Bugoni L (2015) The contrasting feeding ecology of great egrets and roseate spoonbills in limnetic and estuarine colonies. Hydrobiologia 744(1):187–210. 10.1007/s10750-014-2076-1 10.1007/s10750-014-2076-1

[CR16] Buelvas-Soto J, Marrugo-Madrid S, Marrugo-Negrete J (2022) Mercury and lead bioaccumulation in the Dendrocygna autumnalis duck in the subregion of La Mojana, Colombia. Rev MVZ Cordoba 27(1):2337. 10.21897/rmvz.2337 10.21897/rmvz.2337

[CR111] Burger J, Parsons K, Benson T et al (1992) Heavy metal and selenium levels in young cattle egrets from nesting colonies in the northeastern United States, Puerto Rico, and Egypt. Arch Environ Contam Toxicol 23(4):435–439. 10.1007/BF00203806 1444587 10.1007/s10750-014-2076-1

[CR17] Burger J, Gochfeld M (1997a) Lead and neurobehavioral development in gulls: a model for understanding effects in the laboratory and the field. Neurotoxicology 18(2):495–506 9291497

[CR18] Burger J, Gochfeld M (1997b) Risk, mercury levels, and birds: relating adverse laboratory effects to field biomonitoring. Environ Res 75(2):160–172. 10.1006/enrs.1997.3778 9417847 10.1006/enrs.1997.3778

[CR19] Burger J, Gochfeld M (1997c) Age differences in metals in the blood of herring (*Larus argentatus*) and Franklin’s (*Larus pipixcan*) gulls. Arch Environ Contam Toxicol 33(4):436–440. 10.1007/s002449900274 9419263 10.1007/s002449900274

[CR20] Burger J, Gochfeld M (2000) Metals in albatross feathers from midway atoll: influence of species, age, and nest location. Environ Res 82(3):207–221. 10.1006/enrs.1999.4015 10702328 10.1006/enrs.1999.4015

[CR21] Burger J, Gochfeld M (2005) Effects of lead on learning in herring gulls: An avian wildlife model for neurobehavioral deficits. Neurotoxicology 26(4):615–624. 10.1016/j.neuro.2005.01.005 15941590 10.1016/j.neuro.2005.01.005

[CR23] Burger J, Mizrahi D, Tsipoura N, Jeitner C, Gochfeld M (2018) Mercury, lead, cadmium, cobalt, arsenic and selenium in the blood of semipalmated sandpipers (*Calidris pusilla*) from Suriname, South America: age-related differences in wintering site and comparisons with a stopover site in New Jersey, USA. Toxics 6(2):27. 10.3390/toxics6020027 29747411 10.3390/toxics6020027 PMC6027228

[CR24] Burgess NM, Meyer MW (2008) Methylmercury exposure associated with reduced productivity in common loons. Ecotoxicology 17(2):83–91. 10.1007/s10646-007-0167-8 18038272 10.1007/s10646-007-0167-8

[CR25] Carlson JR, Cristol D, Swaddle JP (2014) Dietary mercury exposure causes decreased escape takeoff flight performance and increased molt rate in European starlings (*Sturnus vulgaris*). Ecotoxicology 23(8):1464–1473. 10.1007/s10646-014-1288-5 25030113 10.1007/s10646-014-1288-5

[CR26] Chai L, Li H, Yang Z, Min X, Liao Q, Liu Y, Men S, Yan Y, Xu J (2017) Heavy metals and metalloids in the surface sediments of the Xiangjiang River, Hunan, China: distribution, contamination, and ecological risk assessment. Environ Sci Pollut Res 24(1):874–885. 10.1007/s11356-016-7872-x 10.1007/s11356-016-7872-x 27761857

[CR27] Dauwe T, Bervoets L, Blust R, Eens M (2002) Tissue levels of lead in experimentally exposed zebra finches (*Taeniopygia guttata*) with particular attention on the use of feathers as biomonitors. Arch Environ Contam Toxicol 42(1):88–92. 10.1007/s002440010295 11706372 10.1007/s002440010295

[CR28] De Francisco N, Ruiz Troya JD, Agüera EI (2003) Lead and lead toxicity in domestic and free living birds. Avian Pathol 32(1):3–13. 10.1080/0307945021000070660 12745376 10.1080/0307945021000070660

[CR29] Depew DC, Basu N, Burgess NM, Campbell LM, Evers DC, Grasman KA, Scheuhammer AM (2012) Derivation of screening benchmarks for dietary methylmercury exposure for the common loon (*Gavia immer*): rationale for use in ecological risk assessment. Environ Toxicol Chem 31(10):2399–2407. 10.1002/etc.1971 22865698 10.1002/etc.1971

[CR30] Descalzo E, Camarero PR, Sánchez-Barbudo IS, Martinez-Haro M, Ortiz-Santaliestra ME, Moreno-Opo R, Mateo R (2021) Integrating active and passive monitoring to assess sublethal effects and mortality from lead poisoning in birds of prey. Sci Total Environ 750:142260. 10.1016/j.scitotenv.2020.142260 33182217 10.1016/j.scitotenv.2020.142260

[CR31] Edens FW, Garlich JD (1983) Lead-induced egg production decrease in Leghorn and Japanese quail hens. Poult Sci 62(9):1757–1763. 10.3382/ps.0621757 6634605 10.3382/ps.0621757

[CR32] Espín S, Martínez-López E, Jiménez P, María-Mojica P, García-Fernández AJ (2014) Effects of heavy metals on biomarkers for oxidative stress in Griffon vulture (*Gyps fulvus*). Environ Res 129:59–68. 10.1016/j.envres.2013.11.008 24529004 10.1016/j.envres.2013.11.008

[CR33] Espín S, Andevski J, Duke G, Eulaers I, Gómez-Ramírez P, Hallgrimsson GT, Helander B, Herzke D, Jaspers VL, Krone O, Lourenço R (2021) A schematic sampling protocol for contaminant monitoring in raptors. Ambio 50(1):95–100. 10.1007/s13280-020-01341-9 32399779 10.1007/s13280-020-01341-9 PMC7708607

[CR34] Evers DC, Savoy LJ, Desorbo CR, Yates DE, Hanson W, Taylor KM, Siegel LS, Cooley JH, Bank MS, Major A, Munney K, Mower BF, Vogel HS, Schoch N, Pokras M, Goodale MW, Fair J (2008) Adverse effects from environmental mercury loads on breeding common loons. Ecotoxicology 17(2):69–81. 10.1007/s10646-007-0168-7 17909967 10.1007/s10646-007-0168-7

[CR35] Franson J, Pain D (2011) Lead in birds. In: Nelson Beyer W, Meador JP (eds) Environmental contaminants in biota: interpreting tissue concentrations, 2nd edn. CRC Press, Boca Raton

[CR36] Frederick P, Jayasena N (2011) Altered pairing behaviour and reproductive success in white ibises exposed to environmentally relevant concentrations of methylmercury. Proc R Soc B: Biol Sci 278(1713):1851–1857. 10.1098/rspb.2010.2189 10.1098/rspb.2010.2189 PMC3097836 21123262

[CR37] Frederick PC, Spalding MG, Dusek R (2002) Wading birds as bioindicators of mercury contamination in Florida, USA: annual and geographic variation. Environ Toxicol Chem 21(1):163–167 11804050 10.1002/etc.5620210123

[CR38] Friend M, Franson JC, Ciganovich EA (1999) Field manual of wildlife diseases: general field procedures and diseases of birds. US Geological Survey. https://pubs.usgs.gov/publication/itr19990001

[CR39] Furtado R, Pereira ME, Granadeiro JP, Catry P (2019) Body feather mercury and arsenic concentrations in five species of seabirds from the Falkland Islands. Mar Pollut Bull 149:110574. 10.1016/j.marpolbul.2019.110574 31546110 10.1016/j.marpolbul.2019.110574

[CR40] García-Barrera T, Gómez-Ariza JL, González-Fernández M, Moreno F, García-Sevillano MA, Gómez-Jacinto V (2012) Biological responses related to agonistic, antagonistic and synergistic interactions of chemical species. Anal Bioanal Chem 403:2237–2253. 10.1007/s00216-012-5776-2 22367285 10.1007/s00216-012-5776-2

[CR41] Geens A, Dauwe T, Bervoets L, Blust R, Eens M (2010) Haematological status of wintering great tits (*Parus major*) along a metal pollution gradient. Sci Total Environ 408(5):1174–1179. 10.1016/j.scitotenv.2009.11.029 19959206 10.1016/j.scitotenv.2009.11.029

[CR42] Golden NH, Rattner BA, Cohen JB, Hoffman DJ, Russek-Cohen E, Ottinger MA (2003) Lead accumulation in feathers of nestling black-crowned night herons (*Nycticorax nycticorax*) experimentally treated in the field. Environ Toxicol Chem Int J 22(7):1517–1524 12836976

[CR43] Gómez-Ramírez P, Martínez-López E, María-Mojica P, León-Ortega M, García-Fernández AJ (2011) Blood lead levels and δ-ALAD inhibition in nestlings of Eurasian Eagle Owl (*Bubo bubo*) to assess lead exposure associated to an abandoned mining area. Ecotoxicology 20(1):131–138. 10.1007/s10646-010-0563-3 21076940 10.1007/s10646-010-0563-3

[CR44] Goodale MW, Evers DC, Mierzykowski SE, Bond AL, Burgess NM, Otorowski CI, Welch LJ, Hall CS, Ellis JC, Allen RB, Diamond AW, Kress SW, Taylor RJ (2008) Marine foraging birds as bioindicators of mercury in the Gulf of Maine. EcoHealth 5(4):409–425. 10.1007/s10393-009-0211-7 19277786 10.1007/s10393-009-0211-7

[CR45] Goutte A, Bustamante P, Barbraud C, Delord K, Weimerskirch H, Chastel O (2014) Demographic responses to mercury exposure in two closely related Antarctic top predators. Ecology 95(4):1075–1086. 10.1890/13-1229.1 24933825 10.1890/13-1229.1

[CR46] Hartman CA, Ackerman JT, Herring G, Isanhart J, Herzog M (2013) Marsh wrens as bioindicators of mercury in wetlands of Great Salt Lake: do blood and feathers reflect site-specific exposure risk to bird reproduction? Environ Sci Technol 47(12):6597–6605. 10.1021/es400910x 23692510 10.1021/es400910x

[CR47] Henny CJ, Hill EF, Grove RA, Kaiser JL (2007) Mercury and drought along the lower Carson River, Nevada: I. Snowy egret and black-crowned night-heron annual exposure to mercury, 1997–2006. Arch Environ Contam Toxicol 53(2):269–280. 10.1007/s00244-006-0163-7 17587144 10.1007/s00244-006-0163-7

[CR48] Hoffman DJ, Ohlendorf HM, Marn CM, Pendleton GWP (1998) Association of mercury and selenium with altered glutathione metabolism and oxidative stress in diving ducks from the San Francisco bay region, USA. Environ Toxicol Chem 17(2):167–172. 10.1002/etc.5620170205 10.1002/etc.5620170205

[CR49] del Hoyo J, Elliott A, Sargatal J, Christie DA (1992) Handbook of the birds of the world. Lynx Edicions., Barcelona

[CR50] Hughes MF (2002) Arsenic toxicity and potential mechanisms of action. Toxicol Lett 133(1):1–16. 10.1016/s0378-4274(02)00084-x 12076506 10.1016/s0378-4274(02)00084-x

[CR51] Hutton M, Goodman GT (1980) Metal contamination of feral pigeons *Columba livia* from the London area: Part 1—tissue accumulation of lead, cadmium and zinc. Environ Pollu Ser Ecol Biol 22(3):207–217. 10.1016/0143-1471(80)90015-X 10.1016/0143-1471(80)90015-X

[CR52] Jackson AK, Evers DC, Folsom SB, Condon AM, Diener J, Goodrick LF, McGann AJ, Schmerfeld J, Cristol DA (2011) Mercury exposure in terrestrial birds far downstream of an historical point source. Environ Pollut 159(12):3302–3308. 10.1016/j.envpol.2011.08.046 21903311 10.1016/j.envpol.2011.08.046

[CR53] Janssens E, Dauwe T, Bervoets L, Eens M (2001) Heavy metals and selenium in feathers of great tits (*Parus major*) along a pollution gradient. Environ Toxicol Chem 20(12):2815–2820 11764165 10.1002/etc.5620201221

[CR54] Ji X, Hu W, Cheng J, Yuan T, Xu F, Qu L, Wang W (2006) Oxidative stress on domestic ducks (Shaoxing duck) chronically exposed in a Mercury-Selenium coexisting mining area in China. Ecotoxicol Environ Saf 64(2):171–177. 10.1016/j.ecoenv.2005.03.009 16406582 10.1016/j.ecoenv.2005.03.009

[CR55] Jovanović DA, Marković RV, Teodorović VB, Šefer DS, Krstić MP, Radulović SB, Ivanović Ćirić JS, Janjić JM, Baltić MŽ (2017) Determination of heavy metals in muscle tissue of six fish species with different feeding habits from the Danube River, Belgrade-public health and environmental risk assessment. Environ Sci Pollut Res 24(12):11383–11391. 10.1007/s11356-017-8783-1 10.1007/s11356-017-8783-1 28315055

[CR56] Kamiński P, Kurhalyuk N, Szady-Grad M, Tkachenko H, Kasprzak M, Jerzak J (2008) Chemical elements in the blood of white stork *Ciconia ciconia* chicks in differential Poland regions. Med Biol Sci 22(4):31–37

[CR57] Kanwal S, Abbasi NA, Chaudhry MJI, Ahmad SR, Malik RN (2020) Oxidative stress risk assessment through heavy metal and arsenic exposure in terrestrial and aquatic bird species of Pakistan. Environ Sci Pollut Res 27(11):12293–12307. 10.1007/s11356-020-07649-z 10.1007/s11356-020-07649-z 31993901

[CR58] Keller RH, Xie L, Buchwalter DB, Franzreb KE, Simons TR (2014) Mercury bioaccumulation in Southern Appalachian birds, assessed through feather concentrations. Ecotoxicology 23(2):304–316. 10.1007/s10646-013-1174-6 24420618 10.1007/s10646-013-1174-6

[CR59] Kendall RJ, Scanlon PF (1981) Effects of chronic lead ingestion on reproductive characteristics of ringed turtle doves *Streptopelia risoria* and on tissue lead concentrations of adults and their progeny. Environ Poll Ser Ecol Biol 26(3):203–213. 10.1016/0143-1471(81)90006-4 10.1016/0143-1471(81)90006-4

[CR60] Kim EY, Murakami T, Saeki K, Tatsukawa R (1996) Mercury levels and its chemical form in tissues and organs of seabirds. Arch Environ Contam Toxicol 30(2):259–266. 10.1007/BF00215806 10.1007/BF00215806

[CR61] Kobiela ME, Cristol DA, Swaddle JP (2015) Risk-taking behaviours in zebra finches affected by mercury exposure. Anim Behav 103:153–160. 10.1016/j.anbehav.2015.02.024 10.1016/j.anbehav.2015.02.024

[CR62] Koivula MJ, Eeva T (2010) Metal-related oxidative stress in birds. Environ Pollut 158(7):2359–2370. 10.1016/j.envpol.2010.03.013 20382455 10.1016/j.envpol.2010.03.013

[CR63] Krone O (2018) Lead poisoning in birds of prey. In: Birds of prey. pp. 251–272. Springer International Publishing. 10.1007/978-3-319-73745-4_11

[CR64] Lavoie RA, Baird CJ, King LE, Kyser TK, Friesen VL, Campbell LM (2014) Contamination of mercury during the wintering period influences concentrations at breeding sites in two migratory piscivorous birds. Environ Sci Technol 48(23):13694–13702. 10.1021/es502746z 25380025 10.1021/es502746z

[CR65] Levin R, Zilli Vieira CL, Rosenbaum MH, Bischoff K, Mordarski DC, Brown MJ (2021) The urban lead (Pb) burden in humans, animals and the natural environment. Environ Res 193:110377. 10.1016/j.envres.2020.110377 33129862 10.1016/j.envres.2020.110377 PMC8812512

[CR66] López-Berenguer G, Peñalver J, Martínez-López E (2020) A critical review about neurotoxic effects in marine mammals of mercury and other trace elements. Chemosphere 246:125688. 10.1016/j.chemosphere.2019.125688 31896013 10.1016/j.chemosphere.2019.125688

[CR67] Lucia M, André JM, Gontier K, Diot N, Veiga J, Davail S (2010) Trace element concentrations (mercury, cadmium, copper, zinc, lead, aluminium, nickel, arsenic, and selenium) in some aquatic birds of the southwest Atlantic coast of France. Arch Environ Contam Toxicol 58(3):844–853. 10.1007/s00244-009-9393-9 19763676 10.1007/s00244-009-9393-9

[CR68] Ma Y, Hobson KA, Kardynal KJ, Guglielmo CG, Branfireun BA (2021) Inferring spatial patterns of mercury exposure in migratory boreal songbirds: Combining feather mercury and stable isotope (*δ*^*2*^*H*) measurements. Sci Total Environ 762:143109. 10.1016/j.scitotenv.2020.143109 33162143 10.1016/j.scitotenv.2020.143109

[CR69] Malik RN, Zeb N (2009) Assessment of environmental contamination using feathers of *Bubulcus ibis L*., as a biomonitor of heavy metal pollution, Pakistan. Ecotoxicology 18(5):522–536. 10.1007/s10646-009-0310-9 19418220 10.1007/s10646-009-0310-9

[CR70] Mallory ML, Provencher JF, Robertson GJ, Braune BM, Holland ER, Klapstein S, Stevens K, O’Driscoll NJ (2018) Mercury concentrations in blood, brain and muscle tissues of coastal and pelagic birds from northeastern Canada. Ecotoxicol Environ Contam 157:424–430 10.1016/j.ecoenv.2018.04.004 29655158

[CR71] Markowski M, Kaliński A, Skwarska J, Wawrzyniak J, Bańbura M, Markowski J, Zieliński P, Bańbura J (2013) Avian feathers as bioindicators of the exposure to heavy metal contamination of food. Bull Environ Contam Toxicol 91(3):302–305. 10.1007/s00128-013-1065-9 23912228 10.1007/s00128-013-1065-9 PMC3745608

[CR72] Mateo R, Cadenas R, Máñez M, Guitart R (2001) Lead shot ingestion in two raptor species from Doñana. Spain Ecotoxicol Environ Saf 48(1):6–10. 10.1006/eesa.2000.1996 11161671 10.1006/eesa.2000.1996

[CR73] Mateo R, Beyer WN, Spann JW, Hoffman DJ, Ramis A (2003a) Relationship between oxidative stress, pathology, and behavioral signs of lead poisoning in mallards. J Toxicol Environ Health Part A 66(14):1371–1389. 10.1080/15287390306390 10.1080/15287390306390 12851117

[CR74] Mateo R, Taggart M, Meharg AA (2003b) Lead and arsenic in bones of birds of prey from Spain. Environ Pollut 126(1):107–114. 10.1016/S0269-7491(03)00055-1 12860107 10.1016/S0269-7491(03)00055-1

[CR75] Mirsanjari MM, Sheybanifar F, Arjmand F (1970) The study of forest hara biosphere reserve in coast of Persian gulf and the importance of heavy metal accumulation. Case Study: Feathers of Great Cormorant Nus Biosci. 10.13057/nusbiosci/n060209 10.13057/nusbiosci/n060209

[CR76] Misztal-Szkudlińska M, Szefer P, Konieczka P, Namieśnik J (2011) Biomagnification of mercury in trophic relation of great cormorant (*Phalacrocorax carbo*) and fish in the Vistula Lagoon. Poland Environ Monit Assess 176(1–4):439–449. 10.1007/s10661-010-1595-0 20623377 10.1007/s10661-010-1595-0

[CR77] Murillo-Pacheco J, López-Iborra GM, Escobar F, Bonilla-Rojas WF, Verdú JR (2018) The value of small, natural and man-made wetlands for bird diversity in the east Colombian Piedmont. Aquat Conserv Mar Freshw 28(1):87–97. 10.1002/aqc.2835 10.1002/aqc.2835

[CR78] Naraharisetti SB, Aggarwal M, Ranganathan V, Sarkar SN, Kataria M, Malik JK (2009) Effects of simultaneous repeated exposure at high levels of arsenic and malathion on hepatic drug-biotransforming enzymes in broiler chickens. Environ Toxicol Pharmacol 28(2):213–218. 10.1016/j.etap.2009.04.006 21784005 10.1016/j.etap.2009.04.006

[CR79] Nicholson JK, Osborn D (1984) Kidney lesions in juvenile starlings *Sturnus vulgaris* fed on a mercury-contaminated synthetic diet. Environ Poll Ser Ecol Biol 33(3):195–206. 10.1016/0143-1471(84)90010-2 10.1016/0143-1471(84)90010-2

[CR80] Nyholt K, Jardine TD, Villamarín F, Jacobi CM, Hawes JE, Campos-Silva J, Srayko S, Magnusson WE (2022) High rates of mercury biomagnification in fish from Amazonian floodplain-lake food webs. Sci Total Environ 833:155161. 10.1016/j.scitotenv.2022.155161 35421468 10.1016/j.scitotenv.2022.155161

[CR81] Osičková J, Banďouchová H, Kováčová V, Král J, Novotný L, Ondráček K, Pohanka M, Sedláčková J, Škochová H, Vitula F, Pikula J (2014) Oxidative stress and liver damage in birds exposed to diclofenac and lead. Acta Vet Brno 83(4):299–304. 10.2754/avb201483040299 10.2754/avb201483040299

[CR82] Pain DJ (1989) Haematological parameters as predictors of blood lead and indicators of lead poisoning in the black duck (*Anas rubripes*). Environ Pollut 60(1–2):67–81. 10.1016/0269-7491(89)90221-2 15092391 10.1016/0269-7491(89)90221-2

[CR83] Pain DJ, Mateo R, Green RE (2019) Effects of lead from ammunition on birds and other wildlife: A review and update. Ambio 48(9):935–953. 10.1007/s13280-019-01159-0 30879267 10.1007/s13280-019-01159-0 PMC6675766

[CR84] Perkins M, Lane OP, Evers DC, Sauer AM, O’Driscoll NJ, Edmunds ST, Jackson AK, Hagelin JC, Trimble J, Sunderland EM (2020) Historical patterns in mercury exposure for North American songbirds. Ecotoxicology 29(8):1161–1173. 10.1007/s10646-019-02054-w 31161526 10.1007/s10646-019-02054-w

[CR85] Rutkowska M, Płotka-Wasylka J, Lubinska-Szczygeł M, Różańska A, Możejko-Ciesielska J, Namieśnik J (2018) Birds’ feathers – Suitable samples for determination of environmental pollutants. TrAC Trends Anal Chem 109:97–115. 10.1016/j.trac.2018.09.022 10.1016/j.trac.2018.09.022

[CR86] Salgado J, Shurin JB, Vélez MI, Link A, Lopera-Congote L, González-Arango C, Jaramillo F, Åhlén I, de Luna G (2022) Causes and consequences of recent degradation of the Magdalena River basin, Colombia. Limnol Oceanogr 7(6):451–465 10.1002/lol2.10272

[CR87] Sánchez-Virosta P, Espín S, García-Fernández AJ, Eeva T (2015) A review on exposure and effects of arsenic in passerine birds. Sci Total Environ 512–513:506–525. 10.1016/j.scitotenv.2015.01.069 10.1016/j.scitotenv.2015.01.069 25644847

[CR88] Sánchez-Virosta P, Espín S, Ruiz S, Salminen JP, García-Fernández AJ, Eeva T (2018) Experimental manipulation of dietary arsenic levels in great tit nestlings: Accumulation pattern and effects on growth, survival and plasma biochemistry. Environ Pollut 233:764–773. 10.1016/j.envpol.2017.10.113 29127934 10.1016/j.envpol.2017.10.113

[CR89] Sánchez-Virosta P, Espín S, Ruiz S, Panda B, Ilmonen P, Schultz SL, Karouna-Renier N, García-Fernández AJ, Eeva T (2020) Arsenic-related oxidative stress in experimentally-dosed wild great tit nestlings. Environ Pollut 259:113813. 10.1016/j.envpol.2019.113813 31896481 10.1016/j.envpol.2019.113813

[CR90] Sandoval C, Mora MA, Sericano J, Rech RR (2019) Persistent Organic Pollutants in Livers and Hg in Feathers of Neotropic Cormorants (*Phalacrocorax brasilianus*) from the Trinity River Watershed (Texas, USA). Arch Environ Contam Toxicol 76(3):405–413. 10.1007/s00244-018-00596-4 30623198 10.1007/s00244-018-00596-4

[CR91] Scheuhammer AM (1987) The chronic toxicity of aluminium, cadmium, mercury, and lead in birds: a review. Environ Pollut 46(4):263–295. 10.1016/0269-7491(87)90173-4 15092724 10.1016/0269-7491(87)90173-4

[CR92] Scheuhammer AM (1991) Effects of acidification on the availability of toxic metals and calcium to wild birds and mammals. Environ Pollut 71(2–4):329–375. 10.1016/0269-7491(91)90036-V 15092123 10.1016/0269-7491(91)90036-V

[CR93] Snoeijs T, Dauwe T, Pinxten R, Darras VM, Arckens L, Eens M (2005) The combined effect of lead exposure and high or low dietary calcium on health and immunocompetence in the zebra finch (*Taeniopygia guttata*). Environ Pollut 134(1):123–132. 10.1016/j.envpol.2004.07.009 15572230 10.1016/j.envpol.2004.07.009

[CR94] Sun J, Bustnes JO, Helander B, Bårdsen BJ, Boertmann D, Dietz R, Jaspers VLB, Labansen AL, Lepoint G, Schulz R, Søndergaard J, Sonne C, Thorup K, Tøttrup AP, Zubrod JP, Eens M, Eulaers I (2019) Temporal trends of mercury differ across three northern white-tailed eagle (*Haliaeetus albicilla*) subpopulations. Sci Total Environ 687:77–86. 10.1016/j.scitotenv.2019.06.027 31203010 10.1016/j.scitotenv.2019.06.027

[CR95] Talloen W, Lens L, van Dongen S, Matthysen E (2008) Feather development under environmental stress: lead exposure effects on growth patterns in Great Tits *Parus major*. Bird Study 55(1):108–117. 10.1080/00063650809461511 10.1080/00063650809461511

[CR96] Tartu S, Angelier F, Wingfield JC, Bustamante P, Labadie P, Budzinski H, Weimerskirch H, Bustnes JO, Chastel O (2015) Corticosterone, prolactin and egg neglect behavior in relation to mercury and legacy POPs in a long-lived Antarctic bird. Sci Total Environ 505:180–188. 10.1016/j.scitotenv.2014.10.008 25461020 10.1016/j.scitotenv.2014.10.008

[CR97] Tasneem F, Abbasi NA, Iqbal Chaudhry MJ, Mashiatullah A, Ahmad SR, Qadir A, Malik RN (2020) Dietary proxies (*δ*^*15*^*N, δ*^*13*^*C*) as signature of metals and arsenic exposure in birds from aquatic and terrestrial food chains. Environ Res 183:109191. 10.1016/j.envres.2020.109191 32062182 10.1016/j.envres.2020.109191

[CR98] Tchounwou PB, Yedjou CG, Patlolla AK, Sutton DJ (2012) Heavy Metals Toxicity and the Environment. EXS 101:133. 10.1007/978-3-7643-8340-4_6 22945569 10.1007/978-3-7643-8340-4_6 PMC4144270

[CR99] Tejeda-Benitez L, Flegal R, Odigie K, Olivero-Verbel J (2016) Pollution by metals and toxicity assessment using *Caenorhabditis elegans* in sediments from the Magdalena River, Colombia. Environ Pollut 212:238–250. 10.1016/j.envpol.2016.01.057 26851980 10.1016/j.envpol.2016.01.057

[CR100] Thompson DR, Hamer KC, Furness RW (1991) Mercury Accumulation in Great Skuas *Catharacta skua* of Known Age and Sex, and Its Effects Upon Breeding and Survival. J Appl Ecol 28(2):672. 10.2307/2404575 10.2307/2404575

[CR101] Vizuete J, Pérez-López M, Míguez-Santiyán MP, Hernández-Moreno D (2019) Mercury (Hg), Lead (Pb), Cadmium (Cd), Selenium (Se), and Arsenic (As) in Liver, Kidney, and Feathers of Gulls: A Review. Rev Environ Contam Toxicol 247:85–146. 10.1007/398_2018_16 30413976 10.1007/398_2018_16

[CR102] Wada H, Cristol DA, McNabb FMA, Hopkins WA (2009) Suppressed adrenocortical responses and thyroid hormone levels in birds near a mercury-contaminated river. Environ Sci Technol 43(15):6031–6038. 10.1021/es803707f 19731714 10.1021/es803707f

[CR103] Weech SA, Scheuhammer AM, Elliott JE (2006) Mercury exposure and reproduction in fish-eating birds breeding in the Pinchi Lake region, British Columbia. Canada Environ Toxicol Chem 25(5):1433–1440. 10.1897/05-181R.1 16704079 10.1897/05-181R.1

[CR104] Whitney MC, Cristol DA (2018) Impacts of sublethal mercury exposure on birds: a detailed review. Rev Environ Contam Toxicol 244:113–163. 10.1007/398_2017_4 28710647 10.1007/398_2017_4

[CR105] Wiemeyer SN, Lamont TG, Bunck CM, Sindelar CR, Gramlich FJ, Fraser JD, Byrd MA (1984) Organochlorine pesticide, polychlorobiphenyl, and mercury residues in bald eagle egg–1969-79–and their relationships to shell thinning and reproduction. Arch Environ Contam Toxicol 13(5):529–549. 10.1007/BF01056332 6435548 10.1007/BF01056332

[CR106] Xia P, Ma L, Yi Y, Lin T (2021) Assessment of heavy metal pollution and exposure risk for migratory birds- a case study of Caohai wetland in Guizhou Plateau (China). Environ Pollut 275:116564. 10.1016/j.envpol.2021.116564 33581637 10.1016/j.envpol.2021.116564

[CR107] Zamora-Arellano NY, Ruelas-Inzunza J, García-Hernández J, Ilizaliturri-Hernández CA, Betancourt-Lozano M (2017) Linking fish consumption patterns and health risk assessment of mercury exposure in a coastal community of NW Mexico. HERA 23(6):1505–1521

[CR108] Zapata LM, Bock BC, Palacio JA (2014) Mercury concentrations in tissues of Colombian slider turtles, *Trachemys callirostris*, from northern Colombia. Bull Environ Contam Toxicol 92(5):562–566. 10.1007/s00128-014-1198-5 24458244 10.1007/s00128-014-1198-5

[CR109] Zolfaghari G, Esmaili-Sari A, Ghasempouri SM, Kiabi BH (2007) Examination of mercury concentration in the feathers of 18 species of birds in southwest Iran. Environ Res 104(2):258–265 17307157 10.1016/j.envres.2006.12.002

[CR110] Zrnčić S, Oraić D, Ćaleta M, Mihaljević Ž, Zanella D, Bilandžić N (2013) Biomonitoring of heavy metals in fish from the Danube River. Environ Monit Assess 185(2):1189–1198. 10.1007/s10661-012-2625-x 22527460 10.1007/s10661-012-2625-x

