# Supplementary material for: Elevated Heavy Metal(loid) Blood and Feather Concentrations in Wetland Birds from Different Trophic Levels Indicate Exposure to Environmental Pollutants
Source: Arch Environ Contam Toxicol. 2024 Aug 12;87(2):127–43. doi: 10.1007/s00244-024-01085-7 (PMC11377487; doi:10.1007/s00244-024-01085-7)
Supplement: Supplementary file 1 — Supplementary file1 (DOCX 25 KB) [file 244_2024_1085_MOESM1_ESM.docx]

*Supplementary information*

**Elevated heavy metal(loid) blood and feather concentrations in wetland birds from different trophic levels indicate exposure to environmental pollutants**

Dora Bjedov^1,2^*, Jorge Bernal-Alviz^3^, Jorge Andrés Buelvas-Soto^4^, Laura Ana Jurman^5^, José Luis Marrugo-Negrete^4^

^1^Centre for Ecology, Evolution and Environmental Changes (cE3c) & CHANGE – Global Change and Sustainability Institute, Faculdade de Ciências, Universidade de Lisboa, Campo Grande, 1749-016 Lisbon, Portugal

^2^Departamento de Biologia Animal, Faculdade de Ciências, Universidade de Lisboa, Campo Grande, 1749-016 Lisbon, Portugal

^3^Independent researcher, St. 4A# 3S-81, Jamundí, Colombia

^4^Laboratory of Toxicology and Environmental Management, Department of Chemistry, Water, Applied and Environmental Chemistry Group, Faculty of Basic Sciences, University of Córdoba, Cra. 6 #77-305, Neiva, Montería, Córdoba, Colombia

^5^PrimeVigilance d.o.o.,Oreškovićeva ulica 20/A, 10020 Zagreb, Croatia

*Corresponding author:

Dora Bjedov, PhD

Centre for Ecology, Evolution and Environmental Changes (cE3c) & CHANGE – Global Change and Sustainability Institute, Faculdade de Ciências, Universidade de Lisboa, Campo Grande, 1749-016 Lisbon, Portugal; Departamento de Biologia Animal, Faculdade de Ciências, Universidade de Lisboa, Campo Grande, 1749-016 Lisbon, Portugal

Phone: +385 99 8717 822

Email: [dora.bjedov@gmail.com](mailto:dora.bjedov@gmail.com)

ORCID iD: 0000-0003-1059-7907

**Table SI–1** Spearman ranks correlation coefficients (*r_s_*) between heavy metals mercury (THg), lead (Pb), metalloid arsenic (As) analysed in blood and feather with the body mass of roseate spoonbill, *P. ajaja*. Significant correlation coefficients are noted with * (*P* < 0.05).

|  |  | Blood THg | Blood Pb | Feather THg | Feather Pb | Feather As | Mass |
| --- | --- | --- | --- | --- | --- | --- | --- |
| Blood THg | *r_s_* | 1 |  |  |  |  |  |
| *P* |  |  |  |  |  |  |  |
| Blood Pb | *r_s_* | 0.21 | 1 |  |  |  |  |
| *P* |  | 0.44 |  |  |  |  |  |
| Feather THg | *r_s_* | 0.08 | -0.18 | 1 |  |  |  |
| *P* |  | 0.77 | 0.51 |  |  |  |  |
| Feather Pb | *r_s_* | -0.21 | 0.29 | -0.08 | 1 |  |  |
| *P* |  | 0.44 | 0.30 | 0.77 |  |  |  |
| Feather As | *r_s_* | 0.08 | -0.18 | 1.00 | -0.08 | 1 |  |
| *P* |  | 0.77 | 0.51 | **0.00*** | 0.77 |  |  |
| Mass | *r_s_* | 0.15 | 0.12 | -0.39 | -0.05 | -0.39 | 1 |
| *P* |  | 0.58 | 0.68 | 0.15 | 0.86 | 0.15 |  |

**Table SI–2** Spearman ranks correlation coefficients (*r_s_*) between heavy metals mercury (THg), lead (Pb), and metalloid arsenic (As) analysed in blood and feather with the body mass of black-bellied whistling duck, *D. autumnalis*. Significant correlation coefficients are noted with * (*P* < 0.05).

|  |  | Blood THg | Blood Pb | Blood As | Feather THg | Feather Pb | Feather As | Mass |
| --- | --- | --- | --- | --- | --- | --- | --- | --- |
| Blood THg | *r_s_* | 1 |  |  |  |  |  |  |
| *P* |  |  |  |  |  |  |  |  |
| Blood Pb | *r_s_* | -0.26 | 1 |  |  |  |  |  |
| *P* |  | 0.21 |  |  |  |  |  |  |
| Blood As | *r_s_* | -0.49 | 0.38 | 1 |  |  |  |  |
| *P* |  | **0.02*** | 0.06 |  |  |  |  |  |
| Feather THg | *r_s_* | -0.19 | 0.05 | -0.03 | 1 |  |  |  |
| *P* |  | 0.36 | 0.80 | 0.89 |  |  |  |  |
| Feather Pb | *r_s_* | 0.13 | -0.55 | -0.33 | -0.38 | 1 |  |  |
| *P* |  | 0.53 | **0.01*** | 0.12 | 0.06 |  |  |  |
| Feather As | *r_s_* | -0.47 | -0.15 | 0.32 | 0.25 | 0.26 | 1 |  |
| *P* |  | **0.02*** | 0.49 | 0.12 | 0.23 | 0.21 |  |  |
| Mass | *r_s_* | -0.22 | 0.05 | 0.32 | 0.38 | 0.29 | 0.60 | 1 |
| *P* |  | 0.31 | 0.83 | 0.13 | 0.06 | 0.16 | **0.00*** |  |

**Table SI–3** Pearsons correlation coefficients (*r_s_*) between heavy metals mercury (THg), lead (Pb) and metalloid arsenic (As) analysed in blood and feather with the body mass of neotropical cormorant, *N. brasilianus*. Significant correlation coefficients are noted with * (*P* < 0.05).

|  |  | Blood THg | Blood Pb | Blood As | Feather THg | Feather Pb | Feather As | Mass |
| --- | --- | --- | --- | --- | --- | --- | --- | --- |
| Blood THg | *r_s_* | 1 |  |  |  |  |  |  |
| *P* |  |  |  |  |  |  |  |  |
| Blood Pb | *r_s_* | 0.05 | 1 |  |  |  |  |  |
| *P* |  | 0.93 |  |  |  |  |  |  |
| Blood As | *r_s_* | -0.10 | 0.86 | 1 |  |  |  |  |
| *P* |  | 0.84 | **0.03*** |  |  |  |  |  |
| Feather THg | *r_s_* | 0.06 | 0.04 | 0.40 | 1 |  |  |  |
| *P* |  | 0.91 | 0.94 | 0.43 |  |  |  |  |
| Feather Pb | *r_s_* | 0.69 | 0.54 | 0.33 | 0.30 | 1 |  |  |
| *P* |  | 0.13 | 0.27 | 0.52 | 0.56 |  |  |  |
| Feather As | *r_s_* | 0.49 | 0.46 | 0.66 | 0.60 | 0.46 | 1 |  |
| *P* |  | 0.32 | 0.36 | 0.15 | 0.21 | 0.36 |  |  |
| Mass | *r_s_* | 0.57 | 0.58 | 0.65 | 0.66 | 0.82 | 0.86 | 1 |
| *P* |  | 0.24 | 0.22 | 0.16 | 0.15 | **0.04*** | **0.03*** |  |
